# A plea to merge clinical and public health practices: reasons and consequences

**DOI:** 10.1186/s12913-020-05885-0

**Published:** 2020-12-09

**Authors:** Jean-Pierre Unger, Ingrid Morales, Pierre De Paepe, Michel Roland

**Affiliations:** 1 Department of Public Health, Institute of Tropical Medicine, Nationalestraat 155, B-2000 Antwerp, Belgium; 2 Office de la Naissance et de l’Enfance, French Community of Belgium, Chaussée de Charleroi 95, B-1060 Brussels, Belgium; 3 Département de Médecine Générale, Université Libre de Bruxelles, Route de Lennik, 808, BP 612/1, B-1070 Brussels, Belgium

**Keywords:** Medical professionalism, Medical education, Public health, Health management, Health policy

## Abstract

**Background:**

Revisiting professionalism, both as a medical ideal and educational topic, this paper asks whether, in the rise of artificial intelligence, healthcare commoditisation and environmental challenges, a rationale exists for merging clinical and public health practices. To optimize doctors’ impact on community health, clinicians should introduce public health thinking and action into clinical practice, above and beyond controlling nosocomial infections and iatrogenesis. However, in the interest of effectiveness they should do everything possible to personalise care delivery. To solve this paradox, we explore why it is necessary for the boundaries between medicine and public health to be blurred.

**Main body:**

Proceeding sequentially, we derive standards for medical professionalism from care quality criteria, neo-Hippocratic ethics, public health concepts, and policy outcomes. Thereby, we formulate benchmarks for health care management and apply them to policy evaluation. During this process we justify the social, professional - and by implication, non-commercial, non-industrial - mission of healthcare financing and policies.

The complexity of ethical, person-centred, biopsychosocial practice requires a human interface between suffering, health risks and their therapeutic solution – and thus legitimises the medical profession’s existence. Consequently, the universal human right to healthcare is a right to access professionally delivered care. Its enforcement requires significant updating of the existing medical culture, and not just in respect of the man/machine interface. This will allow physicians to focus on what artificial intelligence cannot do, or not do well. These duties should become the touchstone of their practice, knowledge and ethics. Artificial intelligence must support medical professionalism, not determine it. Because physicians need sufficient autonomy to exercise professional judgement, medical ethics will conflict with attempts to introduce clinical standardisation as a managerial paradigm, which is what happens when industrial-style management is applied to healthcare.

**Conclusion:**

Public healthcare financing and policy ought to support medical professionalism, alongside integrated clinical and public health practice, and its management. Publicly-financed health management should actively promote ethics in publicly- oriented services. Commercialised healthcare is antithetical to ethical medical, and to clinical / public health practice integration. To lobby governments effectively, physicians need to appreciate the political economy of care.

## Background

Accessing professionally delivered health care throughout the full spectrum of individual health care services, from general practitioners (GPs) to university teaching hospitals may be considered a universal human right on the grounds that denying care of this nature amounts to inflicting avoidable suffering, anxiety, and death.

Physicians are especially important in the process of delivering this public duty that is, as duty bearers of this right. To reduce mortality that is amenable to care, they can and ought to contribute to improvements in care quality and accessibility, both in family practice and in hospital medicine:

- GPs are expected to synthesise the patient’s eco-biopsychosocial condition, negotiate with her/him about therapeutic and preventive options^1^ (as per the concepts of person-centred care), as well as facilitate care activities delivered by an inter-professional team.
- Hospital specialists ought to treat the most at-risk patients (where detected by GPs or with emergency condition). As shown in LMICs, GPs and first line health services lose credibility when referred patients cannot easily access hospital services.

Medical professionalism reduces mortality amenable to care and is therefore a key determinant of life expectancy. In East and West Germany, for example, 50–60% and 30–40%, respectively, of the improvements in life expectancy registered in the 1980s were attributable to the health care received [1]. 15.6 million excess deaths from 61 conditions occurred in LMIC in 2016 compared with corresponding mortality from a reference group of 23 high-income countries with strong universal health coverage and good health outcomes. After excluding deaths that could be prevented through public health measures, 55% of excess deaths would have responded to the benefits of health care (either due to receipt of poor-quality care or non-utilisation of health care) [2].

Cultures are made of values, viewpoints that structure knowledge and of the validation of these viewpoints. Professional cultures also include gestures, actions and skills. From a functionalist perspective, medical cultures were designed to promote empathy, reflection, and knowledge transmission and to protect the patient from physicians’ self-serving interests.

Medical cultures and ethics can be seen as inherited wealth that has taken centuries to mature. This wealth was transmitted from generation to generation through education and socialisation, both verbally and by demonstration. In the best cases, this transmission was tailored to the individual physician to enhance her/his effectiveness while building upon his/her personal competencies. To survive down the millennia, Hippocratic ethics had to be updated repeatedly to adjust to technical, socio-cultural, and political changes [3]. In this series, we propose to use the appellation neo-Hippocratic medical ethics to describe practice that is based on the values of “non-maleficence, beneficence, autonomy, and justice … the reference tetrad *par excellence* that physicians and ethicists use to resolve ethical dilemmas. “ [4] The distributive dimension that the quest for ‘justice’ in making clinical and public health decisions appends to the traditional Hippocratic ethics justifies its ‘neo-‘prefix.

### Why should the physicians’ culture evolve today?

Medical culture ought to contribute to a radical change of course in medicine and public health practice.

- Health systems have a responsibility to make sure that medical ethics properly informs the universal right to care, for example, by creating incentives for doctors to provide consistent communication with all their patients [5]. Unfortunately, the commoditisation of healthcare delivery; the advent of commercialised, clinical artificial intelligence; the professionals’ consumerist culture; and the spread of for-profit health financing have all hindered ethical medical practice, with consequences of epidemiological and demographic magnitude. For example,

**Table:** 

a. In a 2014 Tanzanian study, C-section complications accounted for 13% of maternal deaths (95% confidence interval 6.4–23) [6]. This iatrogenesis rises as the rate of caesarean section increases. UNICEF and the World Health Organisation guidelines in 1997 stated that the maximum acceptable rate of C-sections should be 15% [7]. Today, the optimum should be limited to 19% [8]. However, C-section rates were 40.5% in Latin America and the Caribbean region, 32.3% in North America, and 25% in Europe in 2014 [9]. b. Late diagnosis and multi-resistant tuberculosis in LMICs have long been known to arise from poor access to care and unethical medical practices [10]. Physicians tend to treat defaulter tracing and bacilloscopy, rather than X-rays diagnosis, as opportunity costs. This is especially true in vulnerable settings such as slums, where TB prevalence is at its highest due to malnutrition, crowded living conditions and AIDS. This issue concerns the 2.1 billion people who were surviving on less than US$3.10 a day in 2012 [11]. c. Maternal mortality has repeatedly been shown to be dependent on skilled birth attendance. What the wealth of “respectful maternity initiatives” reveals in low-income countries (LICs) is that a level of disrespect, verbal abuse and violence practised by some professionals may be such that fear is an obstacle to skilled birth attendance. d. Finally, consider sex-selective abortion, a problem revealed by Amartya Sen. A 2005 study estimated that over 90 million females were missing from the expected population in Afghanistan, Bangladesh, China, India, Pakistan, South Korea, and Taiwan [12]. In 2010, another study (which also included Bangladesh, Pakistan, China, and India) put this number at slightly less than 38 million [13]. Though at variance, these data reveal the demographic magnitude of a clearly medical-ethical-induced problem.

b. A cultural evolution is needed to tackle the existential (technical, financial and commercial) challenges to the physicians’ professionalism:
  Our era is one of healthcare commoditization. However, the asymmetry that distinguishes a care-giving health professional from a suffering, vulnerable person receiving care could be argued to be the greatest found in any professional practice (lawyers aside). According to neoclassical economics, this should make medical practice, and health care delivery in general, the worst candidate for marketing.
  Governments often underpay physicians and health professionals in public hospitals, thereby accelerating their exodus to parallel private care systems. This, in turn, results in commercial competition, which leads these economic actors to maximise the financial return on their investments in medical practice. In the U.S., the law even establishes a “legally binding fiduciary duty to maximise shareholder profits for the corporation” (Werbel and Carter [14], cited by D. Stuckler [15]).
  The industry generates profits from medical practice while contracting with and directing physicians, at the same time standardising healthcare delivery within narrow confines. Private insurances and governments encourage the industrial-style management of health services (even within public service). The industry competes with doctors for government health funds, whilst investments in new medical technology compete with physicians’ wages.
  Medical culture should strive to strengthen inter-professional teams and the doctors’ ability to lead them. This, contrasts with tendency to de-medicalisation aided and abetted by profit-driven health management. Transferring tasks to nurses could be said to be abusive when the nurses are not properly trained, supervised, and evaluated and their tasks are not properly organised. This occurs when health management is targeted solely or principally at controlling costs. On top of this, AI clinical solutions are being promoted to replace human clinical decision-making - helping to trigger “a crisis in evidence-based medicine” [16].

Notice that AI requires a lot of quantitative information and clear algorithm. It makes thus a useful contribution to regulating fluid balance in intensive care units, for example, but it adds little to clinical decision-making in general practice, where diseases are often seen in early stage, the information available is merely qualitative, and decisions are more complex, because they are highly contextual. Historically, expert systems were initially created to be operated in internal medicine, but they have never been used outside of specific clinical situations. Today, most practical applications are still in their infancy and should be explored and further developed [17]. Radiology is the specialty that has benefited most from advances in AI. Computer-assisted diagnosis of screening mammograms is a valuable example. Recent research in radiology has succeeded in making pneumonia diagnosed by machines more accurate than by humans. In oncology, a computer has recently been able to analyse cat scan images to assess the effectiveness of immunotherapy. Telemedicine makes it possible to monitor a number of parameters (heart rate, sleep level, ECG), with the help of AI, and to communicate information to the doctor in the event of an emergency.

c. Clinicians should be more involved in health management. A study in the UK found that they were the best hospital managers [18]. One possible reason is that, to be effective, health management needs to be intimately connected to clinical work.
d. To the extent that they finance healthcare systems, societies are entitled to expect that clinicians also play a role in preventing (new) public health epidemics, from obesity to burnout, caused by factors ranging from endocrine disruptors to opioids.^2^ Unfortunately, with implications for continuing professional development, ecosystems shaken by industrialisation, are deteriorating so rapidly that doctors’ related knowledge could quickly become out-of-date.
e. At the same time, public health physicians should seek to individualise the delivery of public health programmes at the patient level because, in order to be acceptable, effective, and efficient [19, 20]. Procedures with a strong preventive component (such as TB, AIDS and antenatal consultations alongside under-five and geriatric clinics) ought to be tailored to personal, eco-biopsychosocial risks. This gives physicians an important role in shielding quality of care from the widespread menace of clinical hyper-standardisation and hyper-specialisation in public health programmes. In itself, this is a reason for governments to refrain from de-medicalising public health programmes and international aid.

This article was written for serious consideration by individual practitioners, doctors’ associations, medical faculties and schools of public health. From a normative perspective, it examines that part of the medical (clinical and public health) culture that cannot be addressed by clinical epidemiology and artificial intelligence (AI) and so justify the existence of a human, professional interface in care, healing, prevention and palliation. Hopefully, this exercise will also have relevance for the enhanced professionalism of nurses, physiotherapists, psychologists, dentists, and social workers. While this series as a whole analyses the physician’s mission, status, activities and constraints in the early twenty-first century, this first paper offers doctors a conceptual framework to optimise their impact on population/community health whilst they strive to personalise the clinical conduct of each patient’s care (in line with biopsychosocial and person-centred care delivery concerns). In dissecting medical professionalism, this first paper attempts to place the physician’s guiding values within an environment characterized by heightened tensions between the State, the market, and professional organisations.

The authors expound upon why medical professionalism is essential for population health today and how such practices needs to change in order to remain so, while examining that part of the doctor’s remit that AI does not do or does not do well. The article then provides general guidelines for a joint medical-public health practice and charts the implications of these ideas for ethics, knowledge, education and research, management, financing, and policy.

In addressing professionalism as a medical ideal, an academic programme, and a subject of (continuing) education, this introductory paper studies:

- How medical cultures should evolve in order to adjust to contextual contemporary risks and meet environmental challenges;
- How health policy and management can contribute to, or degrade the professional culture necessary for modern medical practice; and
- What the role exists for public health science in professional healthcare practice.

While answering these questions, this paper aims to define the conditions required for the continued existence of a medical profession promoting moral enterprise at its core.^4^

## Main text

### Benchmarks and standards for contemporary medical, professional practice

#### The challenges of contemporary medical professionalism posed by over-standardised clinical decision-making and artificial intelligence

Currently, AI applied to medicine cannot meet the following quality of care requirements:

- Weighing ethical standards in clinical decision-making, because this also entails philosophical reasoning;
- Delivering eco-biopsychosocial care, because combining health promotion, prevention, curative care, and, in particular, psychological therapy, (and tailoring them to the individual,) is not an exercise suitable for algorithms and standardisation;

c. Avoiding the medicalisation of non-medical issues, because [21], clinically, this is a biopsychosocial decision and collectively, a political and social one.
d. Negotiating clinical decisions with the patient and her/his family, in line with person-centred care because agreeing on therapeutic objectives and conduct (in the framework of goal-oriented care) [22] entails mutual adjustment, egalitarian roles and non-verbal communication [23];

- Using evidence-based medicine (EBM) clinical guidelines critically and creatively, because conflicts of interest among experts often plague clinical guideline design and simultaneous treatment of multiple diseases strains their straightforward implementation [16, 24];
- Acquiring and improving manual, technical, behavioural, and communication skills, because their effective transmission assumes actual physical demonstration and supervised practice;
- Delivering continuous care to ensure that patients complete their treatment or actually use the referral hospitals, because defaulter tracing systems must be complemented by effective doctor-patient dialogue;
- Leading an inter-professional team, because leadership requires motivational skills and sensitivity to local cultural norms.
- Coordinating health care across institutional divides, because that requires negotiation between, among others, GPs and hospital specialists;
- Improving health services’ organisation, because creativity is needed to derive managerial priorities from critical clinical incidents including, for example, “near misses”;
- Collectively developing professional know-how adapted to local epidemiology, because predictive values of signs and lab tests depend on disease prevalence and patients’ mixes that aren’t available to AI designers.
- Reflecting on personal clinical performance to achieve lifelong improvements in providing high quality care, because this exercise applies to all the above-listed domains, and is an insightful process.

The complexity of these tasks justifies the need for a human interface between suffering and health risks on the one hand and their therapeutic and/or prophylactic solution on the other, and mandates that this agent be a high-level, perceptive professional.

Consequently,

- To protect and advance medical professionalism in the modern world, the medical culture should embrace the tasks that AI does not do well (including knowledge and ethics] and thus be a priority for health management, policy, medical education, and research;
- AI ought to be designed and used to support the medical profession and not the reverse. For this, practicing physicians must be included in the development and evaluation processes of AI tools.
- Public health and health management sciences should focus on the delivery of eco-biopsychosocial, person-centred and continuous care; on professional, personal development, on teamwork organisation; on care coordination; on knowledge management and in particular on the prudent use of evidence-based medicine.

Since physicians need to tackle individual and collective health problems simultaneously, our analysis has focused on one important aspect of contemporary medical professionalism, namely, the integration of clinical medicine and public health medicine in practice and theory. Notice that each article in this series addresses a different aspect of this topic.

#### The quality of health care builds on human emotions and sentience

Professional empathy (compassion) is based on a process whereby physicians relate the patient’s suffering, life-goals, risks, and eco-biopsychosocial condition to their own life experiences.

Most medical tasks require the empathy on the part of the physician because, to comply with prescribed treatments, follow lifestyle advice, and use recommended services, patients must be intimately convinced of their relevance and potential effectiveness. That is why doctors and patients need to discuss, negotiate and agree on the determinants of the patient’s suffering and nature of the risks entailed, as endorsed by the person-centred care delivery concept [25].

In general, the same rules ought to apply to negotiating public health programmes with individual communities. But these programmes, that typically carry risks for some in the community and benefits for others are, in general, much more complex. Discussing them in detail is therefore beyond the scope of this paper.

In conducting such negotiations, doctors need to act with self-effacement for the following reasons:

- Professional ethical tenets may be in conflict with each other and these contradictions may give rise to self-serving interests. Without a clear ethical standpoint, physicians tend to react to opposing quality of care considerations, such as a patient’s autonomy and security, or between treatment effectiveness and efficiency, according to distinctly unprofessional considerations, such as whether the patient is rich or not, to optimise their income [25].
- Regulation and control have severe limitations when it comes to healthcare, especially when personal decisions, made in private, are at play [26, 27].

In medicine, exercising empathy and compassion frequently depends on working conditions and income, although less than one might think. In fact, money may even degrade empathy when

- Delivery of care is conceived as commercial transactions since such a commercial relationship assumes the doctor’s *negative*, pecuniary identification with her/his patient: the more money the patient-buyer loses, the more the doctor-seller wins; and
- Clinical decisions are linked to the physicians’ material income – for example the (managed care) ‘pay-for-performance’ technique widely in use in HMOs and PPOs – because it focuses the doctors’ attention on the financial aspects of clinical practice.

This does not mean that physicians should not be adequately paid, but rather that health policies ought to minimise financial incentives in clinical decision-making. They should also make explicit as to how they will enhance the physicians’ *propensity* to altruism – their human, socially [28] and genetically [29, 30] determined altruism.

When invited to list intangible incentives, physicians point to having sufficient time for discussion with colleagues and patients, professional autonomy, teamwork, intellectual progress, or social recognition, but tend to forget professional ethics. *They should not*. If treating medical ethics as an incentive is indeed counter-intuitive, (because, being a feature of quality care, ethics is an output and not an input in healthcare delivery) the assimilation of ethical norms and the adoption of social values can be [31], and must become central factor in physicians’ motivation and behaviour.

That emotions and ethical considerations are conditions for quality of care has structural implications for medical and public health education, management and policy. It is a proposition that is discussed in various points in this paper.

#### How can doctors best adopt a jointly, clinical/public health practice?

Firstly, doctors shouldn’t act as economic agents - since the Hippocratic oath is incompatible with neoclassical economics:

- Doctors should enter the patient’s house solely for the good of the patient. All Hippocratic oath versions include an altruistic benevolence tenet that assumes the physician’s ‘selflessness’, ‘disinterest’ or ‘self-effacement’.
- By contrast, methodological individualism justifies commercial medical practice by hypothesising that humans are beings who generally make rational decisions to optimise their benefit/utility. That they are incompletely informed and restrained in making rational choices (that is, limited by the tractability of the decision problem, the cognitive limitations of the mind, and the time available to make the decision), as the neoclassical economists admit, does not change the case.

Secondly, alongside clinical duties, doctors (*sometimes* in managerial positions) can build and lead teamwork; reflect on practice; contribute behavioural models and promote their colleagues’ professional motivation; coach, educate, train; improve the organisation of their health services; coordinate and evaluate health care; contribute to disease and health risk control; do operational research; and lobby for appropriate health policy design. And, in community-oriented health centres, they can be involved in primary care practices. When physicians inter-relate this to their clinical or disease control practice, they also use their knowledge and experience fully to optimise their impact on the environment. In such a role, doctors could be called “manager-physicians”. Although the term manager is traditionally reserved for persons entrusted with decision-making aimed at achieving their institutions’ predetermined goals most efficiently, our proposed definition assumes that professional ethics should prevail over institutional missions in the doctors’ mind [32].

In fact, any doctor

- *can* adopt the role of “manager-physician” provided that s/he is motivated. This is a rewarding role provided education, research, and good management are professionally valued.
- *should* adopt this role because the opportunity cost of not adopting it can be measured in terms of avoidable suffering and mortality [33].

Doctors ought thus to become “manager-physicians” independently of, or prior to, the adoption of ad hoc institutional policies, without having necessarily been commissioned to act as such: for the sake of a professional ethic designed to optimise doctors’ impact on community health.

#### Knowledge and ethics for integrated medical/public health practices

Over the past 50 years, European policies often encouraged clinicians to introduce interventions that result in collective, positive health spin-offs. These include

- Prescribing antibiotics wisely to reduce nosocomial infections and resistance in hospitals;
- Interpreting lab tests and imaging with semi-quantitative assessment of predictive values [34];
- Shortening the ‘doctor’s delay’,^3^ for example in diagnosing resurgent infectious diseases or in early detection and optimal management of pregnancy bleeding, or of adolescent mental conditions.

To optimise their positive impact on population health, clinicians should systematically broaden their role in the prevention of environmental risks,^4^disease-specific prevention, health education, community-oriented medical practice and health services organisation.

Admittedly, this integrated clinical practice carries a (manageable) risk. While European public health specialists have generally designed disease control programmes incorporated in medical practice in such a way that it protects quality of care, these priorities were not passed on to international cooperation activities in LMICs [20]. The result was large-scale, catastrophic consequences for access to care and its quality [35]. For example, the cooperation agencies set up regional supervisory structures specific to each disease control programme (tuberculosis, AIDS, malaria, maternal health, etc.). In the case of public sector in sub-Saharan Africa, professionals were held accountable to a number of specialised supervisors who each saw only the value of their own programme - to the detriment of a primary health care view including the bio-psychosocial quality of medical care.

Integrated clinical/public health practices call for a new epistemological approach to medical knowledge in order to optimise the impact of clinicians on community health while delivering individualised treatments - and symmetrically, to optimise the impact of public health physicians (and programmes) on individual and family health [36]. This goes for medical ethics: clinical and public health ethics should be merged because medical practice should focus on ‘joined-up’ clinical and public health values [33].

Integrated, medical knowledge and ethics is not only coherent science but also professional because eco-biopsychosocial, person-centred clinical decisions, communication, manual and behavioural skills, reflectivity, provider’s personal development and morals are not easy to standardise but typically situational.

#### Strengthening professional ethics in health systems

Physicians face ethical issues every day: in simply deciding on a lab test (where to determine the cut-off between false positives and false negatives), on the use of medical equipment, or whether and to whom to refer a patient. In striking a balance between their clinical, conviction ethics and managerial, responsibility ethics, physicians, need guidance on ethical clinical decision-making. They need a theory of human resource management designed to promote professionals’ intangible motivations, empathy, and self-effacement which are otherwise part of a physician’s personal history, genetics, identity [37]; prevailing social values and health systems characteristics.

Specifically, such theory would aim to equip physicians who are in managerial and educational functions with psychological guidance to strengthen ethical, medical practice in their hospital or health centre; it would highlight policies that are supportive to this endeavour; and analyse manager-physicians’ needs to promote self-effacement and compassion, I.e.

- A ‘hardware’ that permits human interaction, for example; teamwork, coaching or technical supervision. Notice that to support more intense personal interactions, some health care service structures favour dialogue and cooperation more than others [38, 39].
- A ‘software’, that is to say an interaction model conceived to understand the psychology rooting ethical medical behaviour. The mechanisms that provide the psychic energy to forgo material benefits in clinical decision making link the self-interpretation of the physician’s biography (sometimes called ‘role identity’), his/her (political, philosophical, cultural, religious, etc.) belonging identities, and professional identity.

Some benchmark examples of healthcare management that supports medical professionalism and ethics follow:

- Treating physicians, health professionals, and inter-professional teams not just as the objects of health development strategies, but also their subjects. This is why physicians and health professionals must co-manage non-commercial health services.
- Addressing physicians’ intangible motivations, above and beyond resolving the tension between effectiveness and efficiency that is inherent in any production process: in particular, physicians must be able to decide on the weighting of the quality of care criteria they use in each individual clinical situation.
- Viewing physicians’ personal progress as a health system output and not merely an input to the quality of care. Health services must aim to develop medical professionalism for the physician’s self-esteem and self-realisation.
- Managing professional knowledge, its development and transmission as an organisational priority. In practice, health services shouldn’t focus only on scientific excellence, but also on professional excellence e.g. skills, problem solving capacity and ethics.
- In respect of continuous medical education, this has to involve the transmission of a professional culture in order to advance personal development, rather than merely transmitting competencies.
- Involving health services inter-disciplinary training and education.
- Promoting professional ethics, for example, with ad-hoc inter-professional team debates, clinical case reviews and bottom-up strategies in health services, e.g. care coordination and promotion of professionals’ reflectivity.
- Accepting *sufficient* variability in the implementation of clinical guidelines [40], for example, with opposable decisions: in the French health system, clinical guidelines define what is forbidden in contrast to “command and control” regulations in the UK NHS, where guidelines define what must be done in defined clinical situations.
- Shifting from a disease-oriented paradigm to a clinical ‘goal-oriented’ paradigm, where therapeutic priorities are defined jointly by the doctor and the patient. Goal-oriented decision making is especially important in patients with multi-morbidity, where the multiplicity of problems to be solved and the potential contradictions existing between their solutions make considering ‘least worse’ scenarios inevitable [24].
- Promoting health services system-level thinking, leadership and management. For example patients’ utilization of health services ought to be planned; services should ensure that patients can access the referral structure; services ought to make certain that clinical information accompanies patients through their care journey and the decentralised use of medical technology and drugs should be optimised.

Health and research policies thus ought to recognise the essential difference between the industrial/commercial (generic) and the “bespoke” (or “custom-tailored”), professional/social management of health services:

- The former aims to standardize production processes, thanks to the intervention of high-level technicians, which allows the delegation of tasks to machines and low-skilled workers, and at the end of the day, savings and gains.
- The latter manages production processes as craftsmanship, by which each product care is unique by its eco-biopsychosocial characteristics and the possibility of being negotiated between the doctor and his patient. Such ‘bespoke’ management aims as a priority to improve the professionalism of caregivers, their intangible motivation to practice ethically, their knowledge and their personal development.

The industrial management of health services ought to be banned out of respect for the interpersonal nature of doctor-patient relationships, and the idiosyncrasy of professional development even if the quest for efficiency also features the ‘bespoke management’.

#### Medical heuristics: professional vs. scientific knowledge discovery

Professional knowledge aims to reduce uncertainty in decision-making, increase efficiency in action, relevance in evaluation; stimulate reflexivity; and require the practice to meet ethical standards. Heuristics is defined as any approach to problem solving, learning, or discovery employing a practical methodology that is not guaranteed to be optimal but sufficient for immediate objectives. The heuristic peculiarities of research aimed at discovering new professional knowledge in clinical medicine, public health and health care management distinguish professional medical research from scientific research. Sadly, the latter has completely supplanted the former [41].

Caring (and educating) are idiosyncratic, value-based, person-to-person processes that largely elude probabilistic methodologies. Biomedical sciences largely ignore the implementation of clinical decisions, which are only included within an articulated knowledge system such as (clinical) epidemiology, when the decisions studied can be standardised. For their part, social sciences reach their limits here: descriptive, interpretative methods cannot inform the quality of gestures and speech nor the management of the patient’s suffering and risks in what each of them are, in themselves, unique. Medical, professional research is normative in that it is intended to yield knowledge that is to be given as advice. Without intending to provide advice, the scientific data and descriptions that the researcher publishes offer only random guidance to the practitioner and their relevance is entirely dependent on the any similarities between the environment of the authors and the reader. Strategies are action models to improve clinical and public health practice. They are multi-resource and multi-stage in essence. Action learning and action-research are necessary to conceive, develop and lead them because their very complexity makes trial and error necessary. The validation of a strategy requires repeated experience and its reproducibility assumes a correlation of the environment, which is why the research must sufficiently describe it. To participate to medical action-research, the investigator needs professional proficiency – a requirement that is often difficult in academic settings.

Criteria to assess the relevance of publicly funded medical (clinical and public health) research can be derived from the differences identified between professionally - targeted, and scientific medical research. First, participatory action-research is a research methodology necessary to validate professional knowledge about medical, clinical or public health practice. To support such research, professional teaching methods entail, among other requisites, demonstrations, rotations and/or coaching. Then, with consequences for the development of academic careers and their knowledge content, professional experience ought to be treated as an essential pre-condition for researching clinical, management and public health practice. This is because researchers must be able to identify objectives relevant to professional practice and have sufficient professional credibility to introduce changes within their healthcare organization.

#### Health systems and policy research: objectives, methods and results in health care market evaluation

Since 1980, the commoditization of health care represents the paradigm of health systems reform. The impact of these reforms on healthcare and professional culture and status has been massive [42]:

- Commercialised medical practice follows the privatisation of health financing as insurers and banks take over the management of health services and remunerate physicians, because their profitability depends on the vertical integration of healthcare production lines.
- The commoditisation of health care and its financing reduces access to care, undermines professional ethics and care delivery, and increases mortality and morbidity amenable to care – and probably triggers international migrations [43]. For these reasons not only should commercial medical practice be banned but the appropriate regulation and control of private non-profit and government health services is needed.
- The presence and weight of commercial care financing in total health expenditure should be strictly limited. Publicly-managed health funding ought to be the general rule, as in Europe between 1945 and 2000. Failure to do so could create in HICs the dire health and economic consequences, and the instability that is already visible in most LMICs and in the USA.

Methodological insights for the international comparisons of health systems can be derived from a critical policy analysis. In order to produce knowledge on policies relevant to patients,

- Policy and health systems research needs to mobilise and generate the knowledge that is directly relevant to ethical medical practice and to publicly oriented, non-profit management.
- To study health policies, the methodologies ought to be qualitative/interpretative but include nested quantitative, probabilistic studies.
- They ought to be generally inductive: evaluations in health systems and populations, and public health analysis of clinical practice, care quality and accessibility all are prerequisites enabling the questioning of official, functionalist discourse and the study of the true determinants of public policies through the combined lenses of history, economics, sociology.
- Health systems research ought to be independent of industrial and foundation financing, because interference of vested interest is inevitable. Commercial actors who invest in foundations should not be both the judge of, and subsequently party to, any policy deliberation. Conflicts of interest are inevitable if researchers are asked to evaluate the parties that finance them.

#### Policies for professionally delivered healthcare as a human right

The mission of public policies, health systems and publicly-oriented health services ought to enforce the public’s inalienable right to access ethically, professionally delivered health care in universal health systems. To achieve such objectives, the political leverage of doctors and patients and, in general, of providers and citizen’s coalitions is decisive. However, such coalitions pre-suppose that both patients’ and physicians’ organisations make reciprocal, conceptual and material concessions. To clarify such negotiations, it is necessary to understand the contemporary health care political economy that threatens access to care and at the same time jeopardizes ethics, medical professionalism, and the physician’s social status. In any case, negotiators should rely on public health criteria to explore physicians’ and patients’ common and opposing interests in policies that support the human right to ethical, professional care [44].

For example,

- Physicians’ organisations naturally tend to promote their members’ income and autonomy. To successfully negotiate with patients’ associations, they ought to widen their concerns to include: the population’s access to quality healthcare, the nature of medical practice (professional or commercial), and the promotion of professionalism and medical ethics in healthcare services.
- Patients’ organisations and mutual aid societies (such as the Belgian and French sickness insurance funds) tend to defend access to care but overlook important quality of care issues. They should agree with physicians’ organisations that sufficient time needs to be allocated to consultations and the practitioners' administrative work be strictly limited. Because quality of care incorporates the physician’s ethical motivation, these organisations might easily be persuaded that professional autonomy and the physician’s facilitating role in inter-professional teams is essential to care excellence. Doctors’ remuneration ought to be of an acceptable level in public service; and non-clinical, medical activities (such as continuing medical education; audits and evaluation; coaching and technical supervision; team work; and clinical coordination) should be appropriately financed.

If individual and collective healthcare delivery is to be effective, clinical medicine and public health should be integrated in practice and theory. Health care commoditisation, financing and the emergence of artificial intelligence alter the historical mission of medicine and challenge the status of physicians and other health professionals, with major epidemiological and demographic implications. Self-interest jeopardises professional ethics. Rapid changes in the ecosystem can quickly make whole strands of medical knowledge obsolete. As a result, a weakened medical professionalism undermines the human right to good health care.

## Conclusion

These factors call for changes in the medical culture to support and strengthen those healthcare tasks that AI cannot do, or cannot do well in practice, because AI programmes cannot be empathetic and ethical or deliver biopsychosocial care.

Public policies should sustain the professionalism and intangible motivation of physicians with customised, socially- and professionally-oriented health service management.

To optimise physicians’ personal impact on population health, medical professionalism and ethics should be revisited, with a focus on the accessibility of care, the professionals’ code of ethics and doctor motivation. The same criteria should apply to health care management and policy, medical education and research. All should address the professional’s need for personal development, empathy and altruistic benevolence.

Finally, doctors’ associations concerned with ethical medical issues should join forces with mutual aid societies, and other patients’ organisations concerned with universal access to care, to design sound and humane policy proposals and then use their influence to bring these policies to the attention of political parties and to the forefront of societal debate.

## Data Availability

Data sharing is not applicable to this article as no datasets were generated or analysed during the current study.

## Abbreviations

AI: Artificial intelligence
EBM: Evidence-based medicine
GP: General practitioner
HMO: Health maintenance organization
LMIC: Low and middle income countries
PPO: Preferred providers organization
TB: Tuberculosis

## Footnotes

1 Clinical conducts involve decisions regarding diagnosis, treatment, prevention, utilisation of healthcare services and lifestyle recommendations (life habits, work, diet, exercise, drinking, smoking, etc.).

2 Lack of access to hospital care is a co-factor in opioid mortality, because GPs must then manage pain without being able to influence its aetiology. It is an epidemics co-factor whose importance deserves to be better studied.

3 Patient delay refers to the number of days between the onset of symptoms and the first consultation with a physician. Doctor delay represents the number of days between the first consultation with the doctor and the initiation of a treatment.

4 Among others, the term ‘eco-biopsychosocial care’ implicitly reveals the *essential* continuity of curative and preventive care concepts.
